# Strong delayed negative feedback

**DOI:** 10.3389/fnetp.2024.1399272

**Published:** 2024-06-05

**Authors:** Thomas Erneux

**Affiliations:** Université Libre de Bruxelles, Optique Nonlinéaire Théorique, Bruxelles, Belgium

**Keywords:** network physiology, delayed negative feedback, Mackey-Glass equation, delay differential equation, hopf bifurcation, time periodic oscillations, singular perturbation theory

## Abstract

In this paper, we analyze the strong feedback limit of two negative feedback schemes which have proven to be efficient for many biological processes (protein synthesis, immune responses, breathing disorders). In this limit, the nonlinear delayed feedback function can be reduced to a function with a threshold nonlinearity. This will considerably help analytical and numerical studies of networks exhibiting different topologies. Mathematically, we compare the bifurcation diagrams for both the delayed and non-delayed feedback functions and show that Hopf classical theory needs to be revisited in the strong feedback limit.

## 1 Introduction

The new multi-disciplinary field of Network Physiology concentrates on coordinated network interactions among distinct organs in the human body (Ivanov et al., 2016; Ivanov, 2021; Schöll et al., 2022). These coordinated network interactions are essential to generating distinct physiological states such as wake, sleep and sleep stages, rest and exercise, stress and anxiety, cognition, consciousness and unconsciousness. Disrupting organ communications can lead to dysfunction of individual systems or trigger a cascade of failures leading to a breakdown and collapse of the entire organism, such as sepsis, coma and multiple organ failure. In Refs. (Bashan et al., 2012; Ivanov et al., 2014), the authors considered a dynamical network consisting of ten nodes representing six physiological systems: brain activity (five EEG waves), cardiac, chin muscle tone, leg and eye movements. They observed changes in network topology during different sleep stages (deep, light, and wake). In addition, they recorded time delays between fluctuations in the output signals of one physiological system, such as cardiovascular, and the emergence of corresponding modulations in another, such as the respiratory. According to the authors, the longer the period during which this delay is constant the stronger the coupling between the two systems.

To develop adequate tools for network physiology, recent efforts focused on understanding the network dynamics of coupled excitable or oscillatory units. Traxl et al. (Traxl et al., 2014) study the effects of noise and global coupling strength on coupled oscillators with different network topologies and different node dynamics. They report a general scaling law for the synchronization of such networks. The inclusion of time delays between interacting nodes has a clear impact on the stability of the network. Inspired by leaky integrate-and-fire models for neuronal networks (Politi and Luccioli, 2010), Mafahim et al. (Mafahim et al., 2015) investigate the dynamics of interacting neurons described by

$$\frac{dx_{i}}{dt}=S-\gamma x_{i}+k\underset{j\neq i}{\sum }L_{ij}f\left(t-\tau_{j}\right)$$ (1)

where *k* is the control parameter and *L*_*ij*_ describes the coupling between neurons. Note that *i* = 1, .*N* where *N* is the total number of neurons (nodes). The function *f*(*t*) is a Dirac delta-function. Each neuron moves along the *x* − axis starting at the rest state *x* = 0 and fires when it reaches the threshold *x* = 1. When the neuron fires it forces all the neurons linked to it to make a step ahead or backward by the quantity *k* according to whether *L*_*ij*_ = 1 (excitatory) or *L*_*ij*_ = −1 (inhibitory). The authors highlight the role of inhibitory links in controlling global network dynamics. While considering a simple delayed coupling mechanism between neurons is reasonable for populations of active neurons, delayed nonlinear feedbacks could be more appropriate as communication mechanisms between distinct organs in the body. The mathematical problem then takes the form

$$\frac{dx_{i}}{dt}=g\left(x_{i}\right)+\underset{j\neq i}{\sum }A_{ij}f\left(x_{j}\left(t-\tau_{j}\right)\right)$$ (2)

where *g*(*x*_*i*_) describes the dynamics of *x*_*i*_ in the absence of coupling. The *A*_*ij*_ measures the (small or strong) coupling strengths between nodes. The nonlinear function *f* (*x*_*j*_ (*t* − *τ*_*j*_)) models the delayed feedback of node *j* with respect to node *i*. The complexity of the dynamical problem when *N* > 2 have motivated simplifications which have been explored both analytically and numerically. Networks of delayed coupled Kuramoto oscillators are popular dynamical problems because the state of an oscillator is described by a single angular variable (Laing, 2016; Bick et al., 2020). Another simplification is to consider ring geometries of (unidirectional or bidirectional) coupled nodes (Yuan and Campbell, 2004; Bungay and Campbell, 2007; Ibrahim et al., 2021; Bukh et al., 2023). But the main difficulty remains the fact that we are dealing with coupled delay differential equations (DDEs). If the feedback is strong, however, the feedback function may approach a function exhibiting a threshold nonlinearity which will considerably simplify Eq. 2. In this paper, we consider two delayed negative feedback functions of biological interest and analyze the strong feedback limit. This analysis has never been done and, as we shall demonstrate, Hopf bifurcation theory needs to be revisited.

Negative feedback is one of fundamental mechanisms in cellular networks (Tyson et al., 2003; Tsai et al., 2008; Alon, 2019), which fulfils a variety of functions such as mediating adaptation (Yi et al., 2000; Ma et al., 2009; Ni et al., 2009), stabilizing the abundance of biochemical components (Hasty et al., 2002; Tyson et al., 2003; Alon, 2019), inducing oscillations (Tsai et al., 2008; Elowitz and Leibler, 2000; Kholodenko, 2000; Novak et al., 2007) and decoupling signal and response time (Tyson et al., 2003). Negative feedbacks are shown to be present in many biochemical systems including bacterial adaptation (Yi et al., 2000; Kollmann et al., 2005), mammalian cell cycle (Novak et al., 2010; Ferrell et al., 2011), stress response in yeast (Klipp et al., 2005; Schaber et al., 2012).

A negative feedback control slows of stops a reaction. It may involve a time delay which is needed for signal transduction and transcription, translation and formation of biochemical species (Hoffmann et al., 2002; Börsch and Schaber, 2016). If the delay is too large, however, the control loop loose its landmarks (it does not remember its state so long ago) and exhibit oscillations. The simplest model problem is described by the first order DDE

$$\frac{dx}{dt}=f\left(x\left(t-\tau \right)\right)-bx$$ (3)

where prime means differentiation with respect to time *t*, *x*(*t*) is the state variable, and *x* (*t* − *τ*) is its value at time *t* − *τ*. *τ* > 0 is the delay and *b* > 0 is a constant that measures the rate to equilibrium in the absence of feedback. The nonlinear function *f*(*x*) corresponds to a negative feedback loop: *f* = 1 if *x* (*t* − *τ*) is small (production is activated) and *f* = 0 if *x* (*t* − *τ*) is large (production stops).

We first consider the case

$$f\left(x\right)=-\tanh\left(\kappa x\right)\text{ and }b=0,$$ (4)

and analyze the limit *κ* → *∞*. implying the limit *f*(*x*) = ∓1 as *x* → ±*∞*. Eqs 3, 4 appear in the modeling of delayed coupled cells (Yuan and Campbell, 2004; Bungay and Campbell, 2007) and for a minimal description of ENSO oscillations (Ghil et al., 2008; Keane et al., 2017). Compared to a purely cubic nonlinearity, the negative feedback function 4) saturates as $\left|x\right|$ increases and is a more realistic feedback function.

We next consider the bifurcation diagram of Eq. 3 with the Hill function

$$f\left(x\right)=\frac{1}{1+x^{p}}\text{ and }b>0,$$ (5)

and analyze the limit *p* → *∞* implying the limit *f*(*x*) → 1 − *H* (*x* − 1) where *H*(*y*) is the Heaviside function. Originally, Eqs 3, 5 were modeling the control of hematopoiesis (production of blood cells). Proliferation and maturation of blood cells takes time, so there is a delay, *τ*, between the detection of a deficiency in a circulating population, *x*, and the appearance in the bloodstream of cells to replenish this population (Mackey and Glass, 1977; Glass and Mackey, 1979). Today, it is known as the Mackey-Glass equation and is considered as a reference DDE for any biological process involving a delayed negative feedback [(Fall et al., 2002) p249, (Beuter et al., 2003) p263, (Milton and Ohira, 2014) p236].

The plan of the paper is as follows. In Section 2, we consider the delayed sigmoidal feedback function 4) and determine the bifurcation diagram of the time-periodic solutions. The diagram shows two distinct domains, namely, one close to the Hopf bifurcation point where the amplitude grows parabolically and a larger domain where the amplitude increases linearly. In Section 3, we analyze the delayed Hill feedback function 5). The bifurcation diagram again exhibits two domains with different oscillatory waveforms. Close to the bifurcation point, the small amplitude oscillations quickly change from harmonic to pulsating oscillations. It motivates the analysis of two singular Hopf bifurcations detailed in Section 3.2. In the last section, we emphasize the role of a delayed exponential function appearing in several negative feedback problems and discuss the limit of large delays as another singular limit of physical interest.

## 2 Sigmoidal feedback function

In this section, we analyze Eqs 3, 4 using *τ* as our bifurcation parameter.

### 2.1 Hopf bifurcation analysis

From the linearized theory, we determine the first Hopf bifurcation located at

$$\tau =\tau_{0}\equiv \frac{\pi }{2\kappa }.$$ (6)

We may construct a small amplitude periodic solution near *τ* = *τ*_0_ by using the Lindstedt-Poincaré method (Erneux, 2009; Smith, 2011). We find

$$\begin{array}{ll} x & =2{\left(\frac{\tau -\tau_{0}}{\tau_{0}}\right)}^{1/2}\cos\left(s\right)\\ & \;+\frac{1}{6}{\left(\frac{\tau -\tau_{0}}{\tau_{0}}\right)}^{3/2}\cos\left(3s\right)+O\left({\left(\frac{\tau -\tau_{0}}{\tau_{0}}\right)}^{5/2}\right). \end{array}$$ (7)

By comparing the first two terms in (Eq. 7) in the limit *κ* large, we note that this expansion becomes non uniform if (*τ* − *τ*_0_)/*τ*_0_ = *O* (1), or equivalently, if

$$\tau -\tau_{0}=O\left(\kappa^{-1}\right).$$ (8)

In other words, the domain where the amplitude of the periodic solution increases parabolically as

$$x=\pm 2{\left(\frac{\tau -\tau_{0}}{\tau_{0}}\right)}^{1/2}$$ (9)

is only valid if *τ* − *τ*_0_ ≪ *κ* ^−1^.

### 2.2 Sawtooth oscillations

**FIGURE 1:**
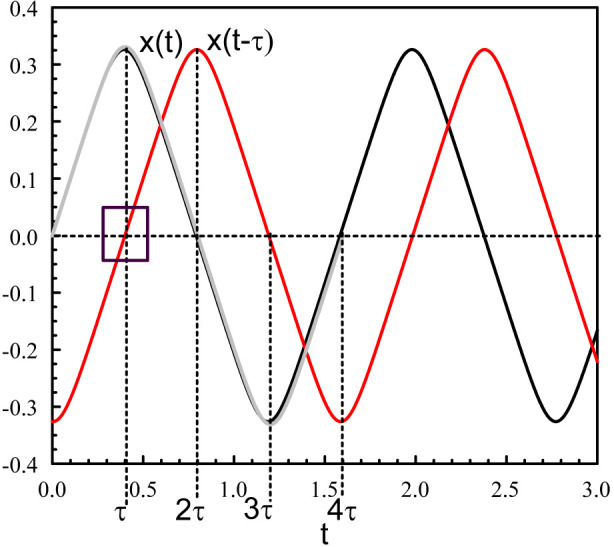
Periodic solution of Eqs 3, 4 for *κ* = 10 and *τ* = 0.4. The figure shows *x*(*t*) (black), *x* (*t* − *τ*) (red), and the leading asymptotic approximation provided by (Eqs 26, 27) (grey). The square shows *x* (*t* − *τ*) ≃ *t* − *τ* when *t* is close to *τ*.

By contrast to our Hopf bifurcation analysis where we were looking for a small amplitude solution and then investigated its behavior for large *κ*, we now seek a periodic solution of arbitrary amplitude but take advantage of the large value of *κ*. A typical numerical solution for *κ* = 10 and *τ* = 0.4 > *τ*_0_ = 0.157 is shown in Figure 1. This solution consists of a succession of straight lines connected at extrema located at *t* = (1 + 2*n*)*τ* (*n* = 0, 1, … ). It motivates to construct an analytical solution by using the method of matched asymptotic expansions (Kevorkian and Cole, 1996; Bender and Orszag, 1999; O’Malley, 2014). The method considers two distinct approximations valid for different intervals of time. The outer approximation, valid for a large subdomain, is obtained by treating the problem as a regular perturbation problem. The inner approximation solves a separate perturbation problem valid in a small subdomain where the outer solution is inaccurate. This area is often referred to as a transition layer. Outer and inner solutions are then combined through a process called “matching” in such a way that a solution for the whole domain is obtained.

### 2.3 Outer solution

Noting that tanh (*κx*) = 1 if *κx* ≫ 1 and tanh (*κx*) = −1 if *κx* ≪ 1, the leading approximation of Eqs 3, 4 satisfies

$$\frac{dx_{0}}{dt}=\left|\begin{array}{c} -1\text{ if }x_{0}\left(t-\tau \right)>0\\ 1\text{ if }x_{0}\left(t-\tau \right)<0 \end{array}\right..$$ (10)

Consequently, *x*_0_(*t*) is alternatively increasing and decreasing as

$$x_{0}=t\left(0<t<\tau \right),$$ (11)

$$x_{0}=\tau -\left(t-\tau \right)\left(\tau <t<3\tau \right),$$ (12)

$$x_{0}=-\tau +\left(t-3\tau \right)\left(3\tau <t<4\tau \right),$$ (13)

and so on. Eq. 10 has been studied by Fridman et al. (Fridman et al., 2002) who showed that only the 4*τ*-periodic solution is stable, whereas the 4*τ*/(4*n* + 1) -periodic oscillations (*n* = 1, 2, … ) are unstable.

### 2.4 Inner solution

We now examine Eqs 3, 4 near *t* = *τ* and *x* = *τ*. To this end, we introduce the variables *s* and *X* defined by

$$t=\tau +\kappa^{-1}s,\;x=\tau +\kappa^{-1}X$$ (14)

We note from Figure 1 (square in the figure) that

$$x\left(t-\tau \right)=t-\tau =\kappa^{-1}s$$ (15)

when *t* is close to *τ*. Eqs 3, 4 then implies that the leading order equation for *X* = *X*_0_ is

$$\frac{dX_{0}}{ds}=-\tanh\left(s\right).$$ (16)

The solution of this equation needs to satisfy matching conditions as *s* → ±*∞*. They are obtained by first introducing (14) into (11). We find

$$x=\tau +\kappa^{-1}X_{0}=t=\tau +\kappa^{-1}s$$ (17)

which implies the condition

$$X_{0}=s\;\left(s\to -\infty \right).$$ (18)

Second, by introducing (14) into (12), we obtain

$$x_{0}=\tau +\kappa^{-1}X_{0}=\tau -\left(t-\tau \right)=\tau -\kappa^{-1}s$$ (19)

which leads to the condition

$$X_{0}=-s\;\left(s\to \infty \right).$$ (20)

The solution of Eq. 16 is

$$X_{0}=-\ln\left(\cosh\left(s\right)\right)+C$$ (21)

where *C* is a constant of integration. We examine the limits *s* → ±*∞* of (Eq. 21) which need to match (18) and (20). We find the conditions.

$$X_{0}\left(s\;\to \;-\infty \right)\to -\ln\left(\exp\left(-s\right)/2\right)+C=s+\ln\left(2\right)+C=s$$ (22)

$$X_{0}\left(s\;\to \;\infty \right)\to -\ln\left(\exp\left(s\right)/2\right)+C=-s+\ln\left(2\right)+C=-s.$$ (23)

Both conditions requires that

$$C=-\ln\left(2\right).$$ (24)

The solution Eq. 21 now is given by

$$X_{0}=-\ln\left(2\cosh\left(s\right)\right).$$ (25)

**FIGURE 2:**
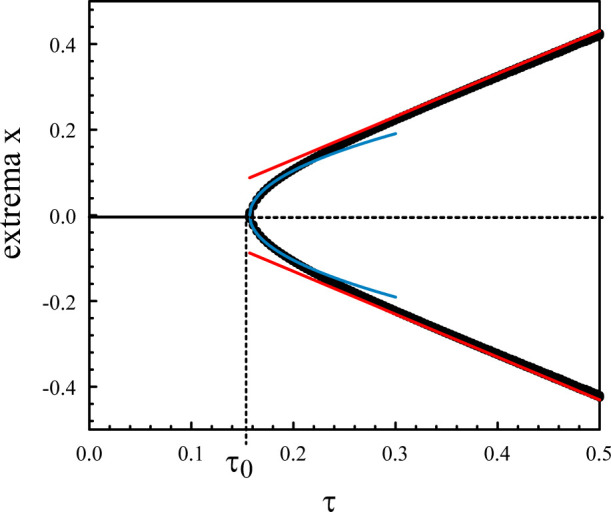
Bifurcation diagram of the periodic solutions of Eqs. 3, 4 for *κ* = 10. The numerical bifurcation diagram of the extrema (black) is compared to Hopf local approximation (9) (blue). The straight lines (red) correspond to the approximation (28).

Figure 2 represents the numerical bifurcation diagram of the periodic solutions for *κ* = 10 together with Hopf local approximation 9) and the large *κ* approximation given by (Eq. 28). Similar inner solutions may be constructed for the other extrema. An uniform solution combining outer and inner solutions leads to.

$$x=\tau -\kappa^{-1}\ln(2\cosh\left(\kappa \left(t-\tau \right)\right)\;\left(0<t<2\tau \right),$$ (26)

$$x=-\tau +\kappa^{-1}\ln(2\cosh\left(\kappa \left(t-3\tau \right)\right)\;\left(2\tau <t<4\tau \right)$$ (27)

and so on. These approximations are compared to the numerical solution (grey line in Figure 1). The reason for such good agreement comes from the fact that the first correction to the leading outer approximation *x* = *x*_0_(*t*) is not *O* (*κ* ^−1^) but much smaller like *O* (exp (−*κ*)). This is because the expansion of tanh (*κx*) as *κx* → ±*∞* is tan(*κx*) = ±1–2 exp (∓*κx*) + as *κx* → ±*∞*. The extrema of the oscillations are obtained from (Eq. 26) and (Eq. 27) at *t* = *τ* and *t* = 3*τ*, respectively:

$$x=\pm \left(\tau -\kappa^{-1}\ln\left(2\right)\right).$$ (28)

## 3 Hill feedback function

By the end of the seventies two independent papers devoted to the development of red blood cells generated considerable mathematical interest. The paper by Wazewska-Czyzewska and Lasota (Wazewska-Czyzewska and Lasota, 1976) and the one by Mackey and Glass (Mackey and Glass, 1977) appeared in 1976 and 1977, respectively. Without knowing each other at that time, these authors published almost simultaneously two models very similar in several points. The one from Wazewska and Lasota is given by (Wazewska-Czyzewska and Lasota, 1976)

$$\frac{dx}{dt}=a\exp\left(-cx\left(t-\tau \right)\right)-bx$$ (29)

where *a*, *b*, and *c* are all positives. The other, today known as one of the two Mackey-Glass equations, is given by Eqs. 3, 5 (Mackey and Glass, 1977; Glass and Mackey, 1979) where *p* > 0 and *b* > 0. The Wazewska-Lasota Eq. 29 was derived from an age structured partial differential equation, and delay was a consequence of its integration. On the other hand, the Mackey-Glass equation Eqs. 3, 5 had been set up directly into a delay differential equation. The nonlinear function 5) is Hill function which is based on the law of mass action for the binding of molecules (Milton and Ohira, 2014). Eqs. 3, 5 has been the source of many numerical and analytical studies. In particular, the limit of a strong feedback (*p* → *∞*) allows to simplify 5) and obtain an analytical approximation. Our objective is to compare its bifurcation diagram with the one obtained numerically from the original DDE with a fixed value of *p*. As we shall demonstrate, the agreement between the two diagrams is excellent except near the Hopf bifurcation points.

Eqs. 3, 5 admit a unique steady state which is unstable if *b*_*H*1_ < *b* < *b*_*H*2_. The critical points *b* = *b*_*H*1_ and *b* = *b*_*H*2_ are Hopf bifurcation points. Their analytical determination is documented at several places (Fall et al., 2002) p249, (Milton and Ohira, 2014), p243 and we briefly detail their conditions. From the steady state equation, we first determine *b* as a function of *x*

$$b=\frac{1}{x\left(1+x^{p}\right)}.$$ (30)

The characteristic equation for the growth rate *λ* is

$$\lambda =-\frac{px^{p-1}}{{\left(1+x^{p}\right)}^{2}}\exp\left(-\lambda \tau \right)-b.$$ (31)

Inserting *λ* = *iω* into Eq. 31, we obtain from the real part a simple expression for *x* ^*p*^ given by

$$x^{p}=-\frac{1}{p\cos\left(z\right)+1}>0$$ (32)

where *z* ≡ *ωτ*. From the imaginary part, we determine the following equation for *τ* as a function of *z* and *b*

$$\tau =-\frac{z}{b\tan\left(z\right)}.$$ (33)

**FIGURE 3:**
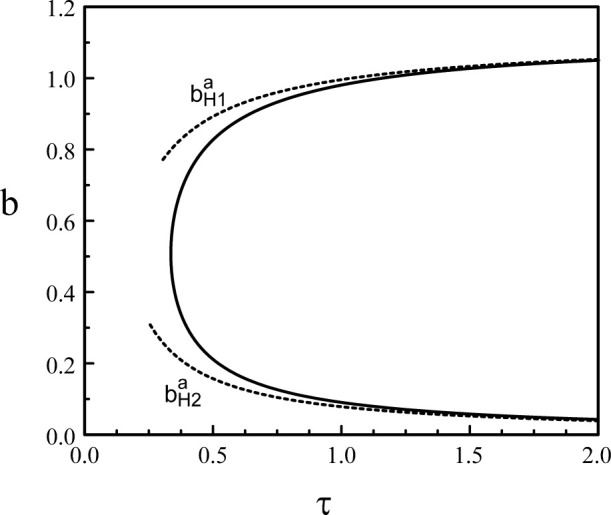
Hopf bifurcation line in the (*τ*, b) parameter space (*p* = 20). $b_{H1}^{a}$ and $b_{H2}^{a}$ denote the large *p* analytical approximations determined in the appendix.

Eqs 30, 32, 33 are the equations defining the Hopf bifurcation in parameter space. Using Eq. 32 for *x* ^*p*^ and determining $x={\left(x^{p}\right)}^{1/p}$ for *x*, we obtain *b* = *b*(*z*) from Eq. 30. The expression for *b* is then introduced in Eq. 33 allowing us to determine *τ* = *τ*(*z*). By continuously increasing *z* (*π*/2 < *z* < *π*), we determine the Hopf bifurcation line in the (*τ*, *b*) parameter space. See Figure 3. The lines denotes by $b_{H1}^{a}$ and $b_{H2}^{a}$ are the large *p* approximations of the upper and lower parts of the Hopf bifurcation line. They are determined in the appendix and their expressions will be useful in the next sections. The lowest Hopf bifurcation point admits the approximation

$$b_{H2}^{a}=\frac{\pi }{2p\tau }.$$ (34)

The approximation of the upper bifurcation point is provided in parametric form (*π*/2 < *z*_0_ < *π* is the parameter).

$$\tau =-\frac{z_{0}}{\tan\left(z_{0}\right)},$$ (35)

$$b_{H1}^{a}=1+\frac{\ln\left(p\right)}{p}-\frac{1}{p}\left(x_{1}+\exp\left(x_{1}\right)\right),$$ (36)

$$x_{1}=-\ln\left(-\cos\left(z_{0}\right)\right)\;\left(\pi /2<z_{0}<\pi \right).$$ (37)

### 3.1 Bifurcation diagrams

By the end of the seventies and early eighties, Mathematicians discovered that piecewise linear (Glass and Mackey, 1979; Mackey and an der Heiden, 1984) or piecewise constant functions (An der Heiden and Walther, 1983) as nonlinearities can make dynamics generated by a scalar delay differential equation accessible, and that one can compute periodic solutions explicitly. In the large p limit, the nonlinear function 5) approaches the function 1 − *H* (*x* − 1) where *H*(*y*) is the Heaviside step function. Consequently, Eqs 3, 5 simplify as

$$\frac{dx}{dt}=-bx+\;\left|\begin{array}{c} \text{0 if }x\left(t-\tau \right)>1\\ 1\text{ if }x\left(t-\tau \right)<1 \end{array}\right..$$ (38)

**FIGURE 4:**
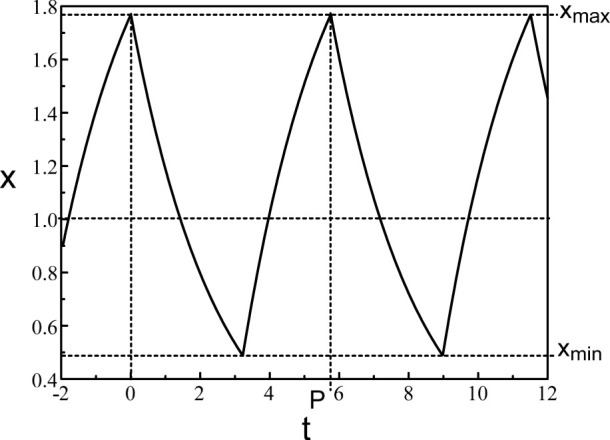
Periodic solution of Eq. 38. *b* = 0.4 and *τ* = 1.8.

A typical periodic solution of Eq. 38 is shown in Figure 4. Eq. 38 consists of a pair of ordinary differential equations which can be solved by the method of steps (An der Heiden and Mackey, 1982). The application of the method is well documented in (Mackey and Glass, 1977; Mackey et al., 1996). The method is also used for a delayed negative feedback problem (Milton, 2003) modeling changes in pupil size. The periodic solution consists of increasing and decreasing exponentials. The extrema of the oscillations are given by.

$$x_{\min}=\exp\left(-b\tau \right),$$ (39)

$$x_{\max}=\left(1-b^{-1}\right)\exp\left(-b\tau \right)+b^{-1}$$ (40)

while the period is

$$P=-b^{-1}\ln\left[\frac{1-bx_{\max}}{1-bx_{\min}}.\frac{x_{\min}}{x_{\max}}\right].$$ (41)

**FIGURE 5:**
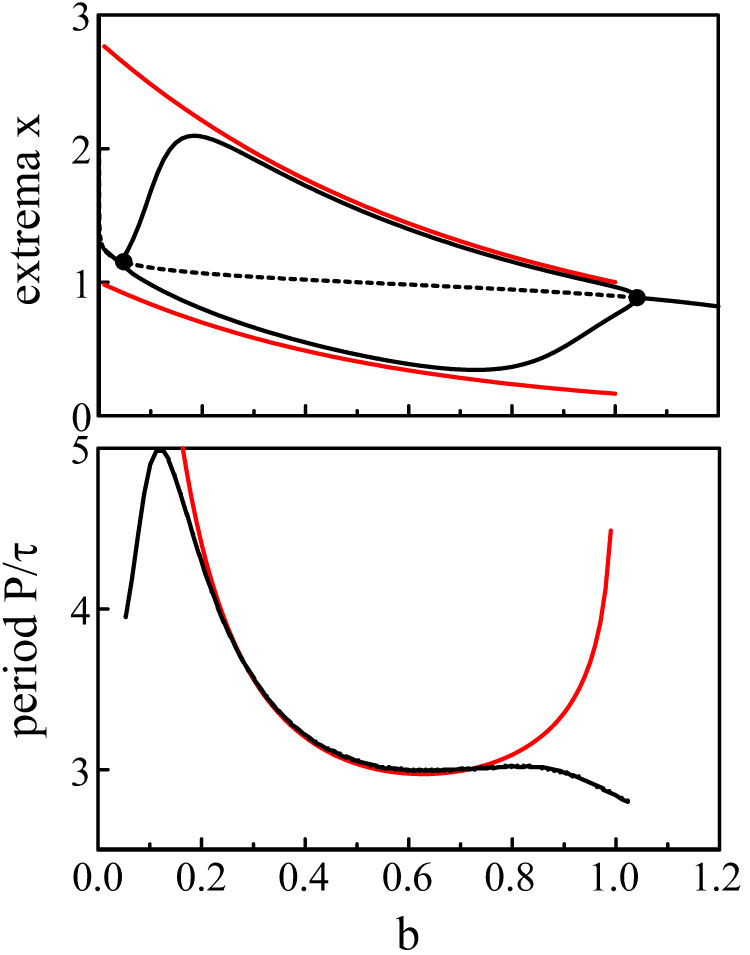
Bifurcation diagram. The fixed parameters are *τ* = 1.8 and *p* = 20. The black lines show the extrema and the period of the limit-cycle oscillations of Eqs 3, 5. The red lines are the approximations of the extrema and period provided by Eqs 39–41.

The expressions (Eq. 39) and (Eq. 40) for the extrema and the Period (Eq. 41) are compared to the numerical bifurcation diagrams obtained from Eqs 3, 5 with *p* = 20. See Figure 5.

The same construction of the solution is proposed in Ref. (Coombes and Laing, 2009). but with *τ* as the bifurcation parameter instead of *b*. The amplitude of the oscillations *x*_max_ − *x*_min_ increases like *τ* and saturates at a fixed value as *τ* → *∞*.

The analytical approximations obtained in the limit *p* → *∞* correctly match the bifurcation branches obtained numerically from Eqs 3, 5 except near the two Hopf bifurcation points where the period becomes infinite. According to Hopf bifurcation theory, the oscillations near the bifurcation point should be nearly sinusoidal and exhibit a fixed period. So how may we understand the radical change of the oscillations from harmonic to pulsating in the vicinity of the two Hopf bifurcation points ? To resolve this problem, we need to take into account the large value of *p* in the construction of a small amplitude solution near each bifurcation points. To this end, we plan to scale the deviation *b* − *b*_*H*_ with respect to *p* ^−1^ and then reexamine the large *p* limit.

### 3.2 Singular hopf bifurcations

**FIGURE 6:**
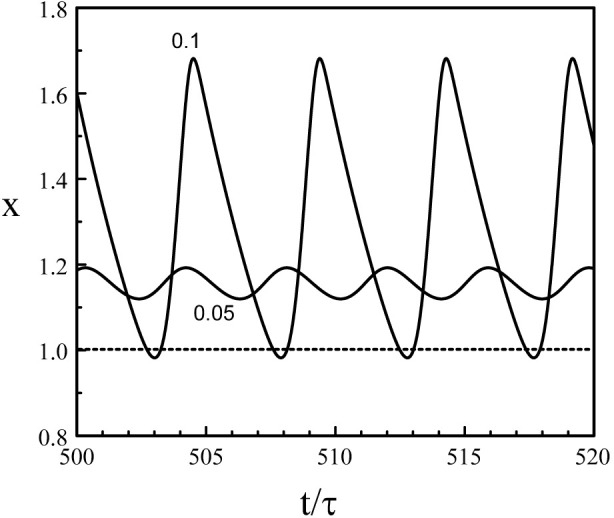
Limit-cycle oscillations close to the Hopf bifurcation point *b* = *b*_*H*2_ = 0.048. *p* = 20 and *τ* = 1.8. The value of *b* is indicated in the figure. The oscillations for *b* = 0.05 are sinusoidal with a period *P* = 3.89 close to the Hopf bifurcation period *P*_*H*2_ ≃ 2*π*/(*π*/2 + *p* ^−1^) = 3.88. The oscillations for *b* = 0.1 exhibit minima slightly below *x* = 1 and the waveform approaches two successive exponentials. The period has increased and equals *P* = 4.88.

We note from Eq. 39 and Eq. 40 that if *b* → 0^+^, *x*_min_ → 1, *x*_max_ → 1 + *τ*, and *P* → *b* ^−1^ ln (1 + *τ*) → *∞*. On the other hand, if *b* → 1^−^, *x*_min_ → exp (−*τ*), *x*_max_ → 1^+^, and *P* → − ln (1 − *b*) → *∞*. Eq. 38 fails to provide the solution of Eqs 3, 5 near *b* = 0 and *b* = 1 because the period *P* become infinite at these points. We also need to realize that our analytical construction of the limit-cycle assumed that *x*(*t*) is sequentially larger and less that 1. This is not the case near the two Hopf bifurcation points where the oscillations remains either above or below 1. Figure 6 shows the limit-cycle oscillations obtained numerically from Eqs 3, 5 for *b* slightly above *b*_*H*2_ ≃ 0.048. The oscillations are sinusoidal for *b* = 0.05 and are clearly above *x* = 1 while the oscillations for *b* = 0.1 have their minima close to *x* = 1.

Our asymptotic theory based on the large value of *p* needs to be revised near the two Hopf bifurcation points. We first consider the lower Hopf bifurcation point *b* = *b*_*H*2_ ∼ 0 for which the analysis is simpler than the case *b* = *b*_*H*1_ ∼ 1.

#### 3.2.1 *b* = *b*_*H*2_ ∼ 0

The analysis of the Hopf bifurcation point detailed in the appendix suggests that *x* ^*p*^ = *O*(*p*) and *b* = *O*(*p* ^−1^). We introduce the new bifurcation parameter *b*_1_ = *O*(1) defined by

$$b=p^{-1}b_{1}$$ (42)

and take into account that *x* ^*p*^ (*t* − *τ*) is an *O*(*p*) large quantity. Eqs 3, 5 then simplifies as

$$x^{\prime }=x^{-p}\left(t-\tau \right)\left(1+O\left(p^{-1}\right)\right)-p^{-1}b_{1}x.$$ (43)

We next introduce the new dependent variable *u* defined by

$$x=1+p^{-1}\ln\left(p\right)+p^{-1}u$$ (44)

where the *p* ^−1^ ln(*p*) term is motivated by the expansion of *x* at the Hopf bifurcation *b* = *b*_*H*2_ (see Appendix). We determine *x* ^−*p*^ (*t* − *τ*) and obtain^1^

$$x^{-p}\left(t-\tau \right)=\frac{1}{p}\exp\left(-u\left(t-\tau \right)\right).$$ (45)

From Eq. 43, we then find that the leading order problem is *O* (*p* ^−1^) and is given by

$$u^{\prime }=\exp\left(-u\left(t-\tau \right)\right)-b_{1}.$$ (46)

**FIGURE 7:**
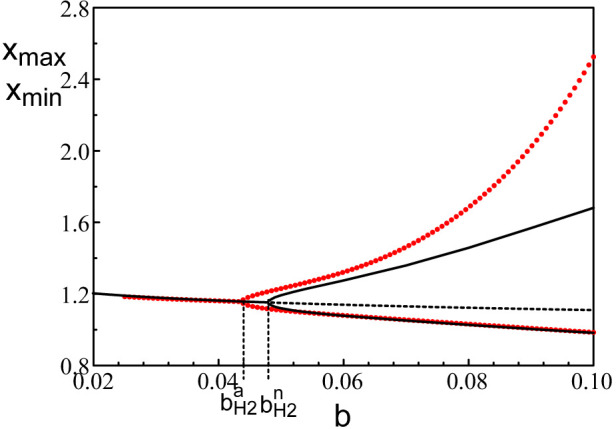
Bifurcation diagrams near *b* = *b*_*H*2_. The black lines correspond to the bifurcation diagram of Eqs 3, 5. The red dots mark the bifurcation diagram of Eq. 46. $b_{H2}^{n}=0.048$ is the Hopf bifurcation point obtained numerically from Eqs 3, 5 and $b_{H2}^{a}=0.044=\;\pi /\left(2p\tau \right)$ is its analytical approximation. The fixed parameters are *p* = 20 and *τ* = 1.8.

Eq. 46 belongs to the family of Wright’s equation (Wright’s equation is Eq. 46 with *b*_1_ = 1). It admits a Hopf bifurcation at *b*_1_ = *π*/(2*τ*). The bifurcation diagram of Eq. 46 is shown in terms of the extrema of *x* in Figure 7 ^2^. The agreement between the minima of the oscillations is excellent but the maxima diverges as soon as *b* > 0.06.

#### 3.2.2 *b* = *b*_*H*1_ ∼ 1

The analysis of the Hopf bifurcation point detailed in the appendix indicates that *x* ^*p*^ = *O* (*p* ^−1^) for the upper Hopf bifurcation branch. Eqs 3, 5 then simplifies as

$$x^{\prime }=1-x^{p}\left(t-\tau \right)+O\left(p^{-2}\right)-bx$$ (47)

We introduce the new dependent variable *u* and new control parameter *b*_1_ = *O*(1) as.

$$x=1-\frac{\ln\left(p\right)}{p}+\frac{1}{p}u,$$ (48)

$$b=1+\frac{\ln\left(p\right)}{p}+\frac{1}{p}b_{1}$$ (49)

where the ln(*p*)/*p* correction term is motivated by the asymptotic expressions of *x* and *b* at *b* = *b*_*H*1_ (see Supplementary Appendix). Using (Eq. 48), we first determine the leading approximation of *x* ^*p*^. We obtain^3^

$$x^{p}\left(t-\tau \right)=\frac{1}{p}\exp\left(u\left(t-\tau \right)\right).$$ (50)

Second, we evaluate *bx* using (Eq. 48) and (Eq. 49). We find

$$bx=1+\frac{1}{p}\left(b_{1}+u\right).$$ (51)

Inserting (Eq. 48), (Eq. 50), and (Eq. 51) into Eq. 47, we find that the leading problem for *u* is *O*(*p* ^−1^) and is given by

$$u^{\prime }=-\exp\left(u\left(t-\tau \right)\right)-\left(u+b_{1}\right).$$ (52)

The steady state solution *u* = *u*(*b*_1_) in implicit form is

$$b_{1}=-\left(u+\exp\left(u\right)\right)$$ (53)

and the conditions for a Hopf bifurcation are.

$$\cos\left(z\right)=-\exp\left(-u\right),$$ (54)

$$\tau =-z/\tan\left(z\right).$$ (55)

The expression (Eq. 49) with (Eq. 53) and *x*_1_ replacing *u* is identical to Eq. 64 in the appendix. Eqs 54, 55 are identical to (66) and (63) in the appendix with *x*_1_ replacing *u* and *z*_0_ replacing *z*.

**FIGURE 8:**
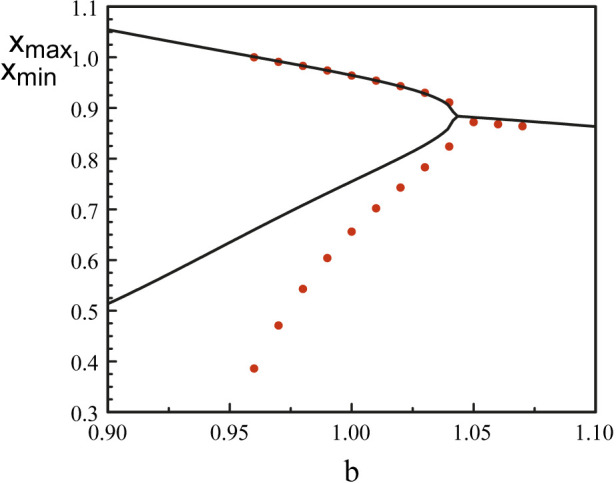
Bifurcation diagrams near *b* = *b*_*H*1_. The black lines correspond to the bifurcation diagram of Eqs 3, 5. The red dots mark the bifurcation diagram of Eq. 52. The analytical Hopf bifurcation point at $b_{H1}^{a}=1.04$ matches the bifurcation point determined numerically. Fixed parameters are *p* = 20 and *τ* = 1.8.

Figure 8 compares the bifurcation diagram of the original equations (Eqs 3, 5) and the bifurcation diagram obtained using the reduced Eq. 52. The agreement between the maxima is excellent but the minima quickly diverges as we deviate from the Hopf bifurcation point.

## 4 Discussion

The new field of network physiology is based on the observation that a healthy body requires good synchronization between different organs. When perturbing elements disturb this equilibrium, many physiological processes are changing from metabolism, immune function to cardiovascular regulation. An example of a simple and well studied network is the circadian network. Circadian rhythms are generated by the autonomous circadian clock, the suprachiasmatic nucleus (SCN), and clock genes that are present in all tissues (Buijs et al., 2016). The SCN times these peripheral clocks, as well as behavioral and physiological processes. Recent studies have shown that frequent violations of conditions set by our biological clock, such as shift work, jet lag, sleep deprivation, or simply eating at the wrong time of the day, may have deleterious effects on health. On the long run, these perturbations are desynchronizing the circadian network.

In this paper, we hypothesize that strong delayed negative feedback loops between elements of the network are essential for a good synchronization. This idea is motivated by the importance of negative feedback in cellular processes. We have considered two delayed negative feedback which have proven to be useful for combined analytical and numerical studies. The limit of strong feedback allows to reduce the delayed function to a function exhibiting a threshold nonlinearity. We have shown that Hopf bifurcation theory needs to be revisited in the case of a strong negative feedback. By treating the Hopf problem as a singular perturbation problem, we determine small amplitude solutions which are quickly changing waveforms as we deviate from the bifurcation point. Like Wazewska and Lasota Eq. 29, the reduced problems for the two Hopf bifurcations of Mackey-Glass equation (Eqs 3, 5) exhibit a delayed exponential nonlinearity. The latter also appeared in a minimal model for periodic or episodic star formation (Alice et al., 2008).

**FIGURE 9:**
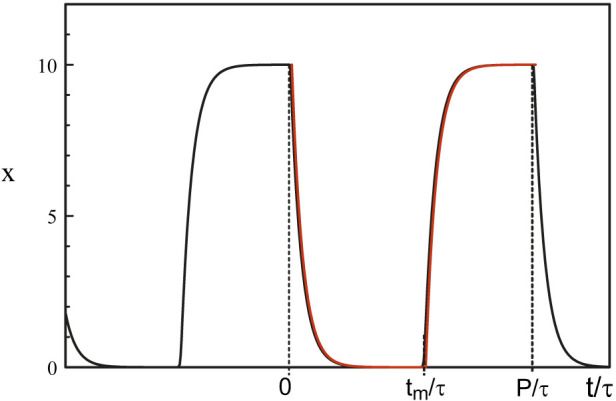
Periodic solution obtained numerically from Eqs 3, 5. The parameters are *τ* = 100, *b* = 0.1, and *p* = 20. The red curves are the large p approximations obtained from solving Eq. 38. Fast transition layers appears at *t* = 0, *t* = *t*_*m*_ and *t* = *P*.

The singularity of the Hopf bifurcation caused by the strong feedback limit is not the only one of physical interest. The limit of large delay is another case where harmonic oscillations quickly become 2*τ* − periodic square-waves as we deviate from the Hopf bifurcation point (Erneux et al., 2004). From the analytical solution of Eq. 38, we note that the square-wave is switching from 0 to *b* ^−1^ through fast transition layers consisting of decaying exponentials. Figure 9 shows the time-periodic solution of Mackey-Glass equations Eqs 3, 5 for a large value of the delay *τ*.

## Footnotes

1

$\ln\left(x^{-p}\right)=-p\ln\left(x\right)=-p\ln\left(1+p^{-1}\ln\left(p\right)+p^{-1}u\right)=-p\left[\left(p^{-1}\ln\left(p\right)+p^{-1}u\right)\right]$ and thus: *x* ^−*p*^ = *p* ^−1^ exp (−*u*)

2 In our simulations, we consider the logistic equation equivalent to Eq. 46 after the change of variables *u* = − ln(*v*). It is given by $v^{\prime }=v\left(b_{1}-v\left(t-\tau \right)\right).$ and *x* = *x*(*v*) then is $x=1+\ln\left[{\left(p/v\right)}^{1/p}\right].$

3

$\ln\left(x^{p}\right)=p\ln\left(x\right)=p\ln\left(1-\frac{\ln\left(p\right)}{p}+\frac{1}{p}u\right)=p\left[-\frac{\ln\left(p\right)}{p}+\frac{1}{p}u\right]$ = − ln(*p*) + *u* and thus: *x* ^*p*^ = *p* ^−1^ exp(*u*)

## Appendix

### The large *p* limit of the Hopf bifurcation points of Eqs. (3) and (5)

In this appendix, we determine the large *p* limit of the upper and lower parts of the Hopf bifurcation line *b* = *b*(*τ*) shown in Figure 3. The conditions for the Hopf bifurcation are provided by the steady state equation (30) and Eqs. (32), (33).

### The lower Hopf bifurcation *b* = *b*_*H*2_ ≪ 1

Numerical simulations suggest that *x* ^*p*^ = *O*(*p*) and *x* ∼ 1 for the lower Hopf bifurcation branch. It motivates to seek a solution for *x* of the form

$$x=1+p^{-1}\ln\left(p\right)+p^{-1}x_{1}+\cdots$$
(56)

where the *p* ^−1^ ln(*p*) correction term is needed when we determine *x* ^*p*^ and *x*_1_ = *O*(1). We find^4^

$$x^{p}=p\exp\left(x_{1}\right)$$
(57)

as the leading approximation. From Eqs. (30) and (32), we then determine *b* and *z* ≡ *ωτ* as

$$b=\frac{1}{p}\exp\left(-x_{1}\right),$$
(58)

$$z=\frac{\pi }{2}+p^{-1}+p^{-2}\exp\left(-x_{1}\right).$$
(59)

Last, we evaluate *τ* from (33) and obtain

$$\tau =\frac{\pi }{2pb},$$
(60)

or equivalently,

$$b=b_{H2}^{a}\equiv \frac{\pi }{2p\tau }.$$
(61)

### 5.2 The upper Hopf bifurcation *b* = *b*_*H*1_ ∼ 1

Numerical simulations now suggest that *x* ^*p*^ = *O*(*p* ^−1^) and *x* ∼ 1 for the upper Hopf bifurcation branch. We seek a solution for *x* of the form

$$x=1-p^{-1}\ln\left(p\right)+p^{-1}x_{1}+\cdots$$
(62)

where the −*p* ^−1^ ln(*p*) correction term is needed when we determine *x* ^*p*^ and *x*_1_ = *O*(1). We obtain^5^

$$x^{p}=p^{-1}\exp\left(x_{1}\right).$$
(63)

From Eq. (30), we then determine *b* as

$$b=b_{H1}^{a}\equiv 1+p^{-1}\ln\left(p\right)-p^{-1}\left(x_{1}+\exp\left(x_{1}\right)\right).$$
(64)

Inserting

$$z=z_{0}+p^{-1}z_{1}+\cdots$$
(65)

into Eq. (32), we find that the leading equation is cos(*z*_0_) = − exp(−*x*_1_). It then provides an expression for *x*_1_ = *x*_1_(*z*_0_) given by

$$x_{1}=-\ln\left(-\cos\left(z_{0}\right)\right)\;\left(\pi /2<z_{0}<\pi \right).$$
(66)

Last, we evaluate *τ* from Eq. (33) and find

$$\tau =-\frac{z_{0}}{\tan\left(z_{0}\right)}.$$
(67)

In summary, the large *p* limit of the upper Hopf bifurcation branch shown in Figure 3 is provided in parametric form by Eq. (64) with *x*_1_ = *x*_1_(*z*_0_) determined from (66), and by Eq. (67) (*z*_0_ is the parameter).
